# Bee-Keeping as a Relaxation

**Published:** 1911-07-22

**Authors:** 


### The Practitioner's Relaxations

### BEE-KEEPING AS A RELAXATION.

The doctor who spends a sunny spring afternoon poring over his medical literature in a small, smoky room may be filling his head with facts, but is depriving himself of the power to make use of them. Let him spend some of the day in the open air, with his mind concentrated on some other aspect of life, and in the evening he will find that his head is clearer and his brain more receptive for the perusal of his medical journals. We have a wealth of opportunity for choosing a hobby, and what appeals to one may not appeal to another. Again, how much we miss through ignorance of the interest attaching to some really choice and profitable hobbies! Until I came to study the one to which I wish to draw your attention I had absolutely no idea of the intense interest attaching to it, and could I but induce some of my professional colleagues to start bee-keeping, for that is my hobby, I am sure they would agree with me that few hobbies can produce so much interest and profit as this one.

It was when I was a student that I first started bee-keeping on a small scale, and through the kindness of friends I was given a present of a bee-hive and all that was required to start on what I thought would turn out a very painful fiasco. How well I remember buying my first swarm of bees, and the distance at which I stood while *somebody else* hived them for me, but when I was asked to come over to the hive and play catch with a handful of bees, I made for the house. However, having put my hand to the plough, I was not going to turn back, so I again ventured forth, with my head enveloped in a large veil, with tight cuffs to prevent the bees getting up my sleeves, and with a pair of gloves on. In this attire I actually walked up to the hive and held my hand open to have a handful of bees put in, and thenceforward my interest was awakened. I soon dispensed with gloves as I found them too clumsy to work in, and though I have had hundreds of bees walking over my hands I have had remarkably few stings. I may here mention a point of interest to the medical man: the bee expert who started me on bee-keeping told me that before he started he used to suffer very much from rheumatism and had had several acute attacks, but since starting had never suffered another rheumatic pain, and this he ascribes to the bees' sting.

There are three classes of bees in the hive. The queen bee is placed in a class by herself, the worker bees, and the drones. Already we have touched upon a most interesting point, for all the eggs laid by the queen are similar and yet it rests with the other bees to decide which of the three classes the embryo bee is to join. Should it be springtime and the hive be overcrowded, the bees will select several of these eggs and will build around them cells much larger than the others; these grubs will be fed with a special food, and after fifteen days hatch out as queen bees. Again, should a drone or male bee be desired another form of cell is built, another food is used, and after twenty-one days the drone bee is hatched. Yet again another form of cell, another kind of food, and within twenty-five days a worker bee (imperfect female) comes forth. Each of these three classes has its own special function to perform; that of the queen is confined entirely to laying eggs, and under favourable circumstances she will lay about two thousand per day. The worker-bee, on the other hand, has no egg-laying to do, but produces wax with which to build the cells. She has also to collect the food for the young and act as their nurse, and it is to her alone that the duty of honey-gathering is entrusted. The drones, or male bees, unlike the human males, do none of the work. One of their most important functions is to fertilise the queen-bee, which is only done once, and henceforward the queen is capable of laying eggs producing bees in any of the classes, though before being fertilised she could only lay eggs that would produce drones. The drones also act as police force to the hive and see that the workers do their work thoroughly; and also assist in keeping up the temperature of the hive. When autumn comes and the bees are preparing themselves for the winter, they set upon the drones and kill them all, as they are then useless in the hive and would only eat up the winter supply of honey. The cells are hexagonal, exact in every measurement; they are not placed at right angles to the foundation, but slope downwards so as to form a pocket into which the eggs and honey can be safely placed. The hive consists of two parts, the body and the super. The body forms the home of the bees and it is here the queen-bee lays her eggs, and by an ingenious arrangement she is prevented from entering the super, which is kept for the reception of the sections to be filled with honey. Upwards of 120 pounds of honey has been stored in a single hive in one summer, so it will be realised that bee-keeping is not merely pleasurable, but profitable.

I feel I have given a very inadequate outline of this fascinating hobby, but I may have said sufficient to inspire others to start an apiary of their own; should they wish to know more of its charms may I advise them to read Maeterlinck's "Life of the Bee," published by George Allen.

